# pH‐sustaining nanostructured hydroxyapatite/alginate composite hydrogel for gastric protection and intestinal release of *Lactobacillus rhamnosus* GG

**DOI:** 10.1002/btm2.10527

**Published:** 2023-04-19

**Authors:** Jihyun Kim, Shwe Phyu Hlaing, Juho Lee, Dongmin Kwak, Hyunwoo Kim, Aruzhan Saparbayeva, In‐Soo Yoon, Eunok Im, Yunjin Jung, Jin‐Wook Yoo

**Affiliations:** ^1^ College of Pharmacy and Research Institute for Drug Development Pusan National University Busan South Korea

**Keywords:** alginate, composite hydrogel, gastric pH resistance, hydroxyapatite, intestinal delivery, probiotics

## Abstract

The gut microbiome is closely linked to gastrointestinal health and disease status. Oral administration of known probiotic strains is now considered a promising therapeutic strategy, especially for refractory diseases such as inflammatory bowel disease. In this study, we developed a nanostructured hydroxyapatite/alginate (HAp/Alg) composite hydrogel that protects its encapsulated probiotic *Lactobacillus rhamnosus* GG (LGG) by neutralizing hydrogen ions that penetrate the hydrogel in a stomach without inhibiting LGG release in an intestine. Surface and transection analyses of the hydrogel revealed characteristic patterns of crystallization and composite‐layer formation. TEM revealed the dispersal of the nanosized HAp crystals and encapsulated LGG in the Alg hydrogel networks. The HAp/Alg composite hydrogel maintained its internal microenvironmental pH, thereby enabling the LGG to survive for substantially longer. At intestinal pH, the encapsulated LGG was completely released upon disintegration of the composite hydrogel. In a dextran sulfate sodium‐induced colitis mouse model, we then assessed the therapeutic effect of the LGG‐encapsulating hydrogel. This achieved intestinal delivery of LGG with minimal loss of enzymatic function and viability, ameliorating colitis by reducing epithelial damage, submucosal edema, inflammatory cell infiltration, and the number of goblet cells. These findings reveal the HAp/Alg composite hydrogel as a promising intestinal‐delivery platform for live microorganisms including probiotics and live biotherapeutic products.

## 1 INTRODUCTION

Intestinal delivery of live microorganisms such as probiotics to gut microbiome with minimal loss of viability is closely related to obtaining desired health benefits or therapeutic effects to the host. Microbiome‐based therapeutics are considered a promising therapeutic alternative for treating refractory diseases such as inflammatory bowel disease that are difficult to cure with traditional small‐molecule medicine.^1^, ^2^ Certain microorganisms, such as probiotic strains, which were once regarded as food or food supplements, have gained attention as therapeutic agents.^3^ The United States Food and Drug Administration (FDA) has created a category for live biotherapeutic products, which includes live microorganisms that can be used to prevent, treat, and cure disease.^4^ In 2021, therapy for *Clostridium difficile* infection, using live *firmicutes* spores, passed FDA phase‐3 approval.^5^ Accordingly, there has been a rapid increase in expectations for microbiome therapeutics based on live microorganisms once regarded as probiotic strains in industry and academia.

Encapsulation of probiotics by using alginate (Alg) hydrogels is the most widely used method due to mild gelation process, pH‐sensitive disintegration, low cost, and versatile application. However, owing to the porous network and hydrophilic nature of Alg hydrogel, it does not sufficiently maintain probiotic viability in the stomach. Furthermore, Alg hydrogels are mechanically unstable in the presence of monovalent ions, presenting another difficulty in their application. This can be addressed by adding surface coatings to Alg hydrogel beads using cationic materials such as chitosan. Reducing the pore size, and consequently maintaining probiotic cytoplasmic pH, enhances probiotic survival.^6^ The gastric survival of probiotics in surface‐coated hydrogels could potentially be enhanced by increasing the number of coating layers on the hydrogel; however, the coating processes are time‐consuming, and probiotic survival declines during storage, possibly due to the effects of cationic coating materials.^7^, ^8^ Composite hydrogels, using materials such as carboxymethyl cellulose,^9^ xanthan gum,^10^ locust bean gum,^11^ nanocellulose,^12^ and clay,^13^ could enhance encapsulated‐probiotic survival at gastric pH. However, the increased viscosity of the mixed slurry could present difficulties during manufacturing, especially during the extrusion process. Further, composite hydrogels are subject to strong molecular interactions, potentially delaying their disintegration and release of encapsulated probiotics in the host intestine.

The physical barrier established in Alg‐based delivery systems, such as coating and composite hydrogel, may reduce their efficacy in probiotic intestinal delivery by interfering with the fabrication of the probiotic‐loaded hydrogel, protection in the stomach, and release in the intestine. Physical barriers based on additional coating materials or composite hydrogel networks may passively delay penetration by gastric fluid and thus extend probiotic survival. However, although physical barriers enhance resistance to gastric pH, they can easily interfere with other critical aspects of the probiotic intestinal‐delivery system. Few studies have addressed chemical barrier systems comprising pH buffering agents in Alg‐based systems. Alg hydrogels are based on ionically crosslinked networks that can be easily disturbed by calcium‐chelating agents or monovalent ions.^14^, ^15^ Most buffering agents comprise conjugate acid–base pairs that contain monovalent ions; when these monovalent ions dissociate, the hydrogel cannot sufficiently protect the encapsulated probiotics from penetrating gastric fluid due to the disturbed hydrogel network.

Hydroxyapatite (HAp), a bioceramic material, has pH‐modulating properties owing to its surface functional groups.^16^ Under stomach pH conditions, HAp consumes massive amounts of hydrogen ions as it dissolves, liberating Ca^2+^ ions. Ca^2+^ ion replenishment via ethylenediaminetetraacetic acid has been reported to enhance the gastric survival of encapsulated probiotics by reinforcing hydrogel networks.^17^ HAp, which occurs naturally in bones and teeth, is biocompatible and does not damage live microorganisms.^18^ The inclusion of HAp in Alg hydrogels has been considered for the controlled release of drugs and for osteoblast‐based bone‐tissue recovery.^19^, ^20^ Nonetheless, no prior studies have examined HAp/Alg composite hydrogels for the development of intestine‐targeted probiotic delivery, in our knowledge.

As HAp can both store Ca^2+^ ions and modulate pH, we hypothesized that an HAp‐based delivery system may enhance gastric survival of probiotics by maintaining microenvironmental pH. In this study, we developed a nanostructured HAp/Alg composite hydrogel via ionic gelation and crystallization. The hydrogel structures were analyzed by scanning and transmission electron microscopy (SEM and TEM). Internal pH changes in the hydrogels were observed by using a pH indicator. Probiotic gastric survival and intestinal release were assessed under simulated in vitro conditions, and gastrointestinal hydrogel behavior was tracked in vivo. Furthermore, we evaluated in vivo therapeutic efficacy in a dextran sulfate sodium (DSS)‐induced colitis mouse model.

## 2 MATERIALS AND METHODS

### 2.1 Materials

*Lactobacillus rhamnosus* GG (LGG) was obtained from The Korean Collection for Type Cultures (KCTC 5033; Jeongeup, South Korea). De Man, Rogosa, and Sharpe (MRS) broth and peptone were purchased from BD Biosciences (San Jose, California). Sodium alginate (molecular weight, 80,000–120,000 Da; mannuronate:guluronate ratio, 1:56), sodium chloride, sodium citrate tribasic, sodium hydroxide, bromophenol blue (BPB), vancomycin hydrochloride, and vegitone MRS agar were purchased from Sigma‐Aldrich (St. Louis, Missouri). Calcium chloride dihydrate was purchased from Kanto Chemical Co. Ltd. (Tokyo, Japan). Agar was purchased from Yakuri Pure Chemicals Co. Ltd. (Kyoto, Japan). Diammonium hydrogen phosphate, dibasic potassium phosphate, and hydrochloric acid were purchased from Junsei Chemical (Tokyo, Japan). IR780 iodide was purchased from Alfa Aesar (Ward Hill, Massachusetts). All reagents and solvents used were of the highest grade commercially available.

### 2.2 LGG preparation

For the starter culture, a single LGG colony was inoculated into MRS medium and cultured for 12 h. Then, 5 mL of the starter culture was transferred to 195 mL MRS medium and cultured for 12 h (37°C, with shaking at 220 rpm). LGG was harvested and washed twice with 0.1% peptone, followed by redispersion in deionized distilled water (ddH_2_O) for encapsulation.

### 2.3 Encapsulation of LGG in the HAp/Alg composite hydrogel

Hydroxyapatite/alginate (HAp/Alg) composite hydrogels were fabricated using a modified version of a previously reported method.^21^ Briefly, 3 g sodium alginate was dissolved in 190 mL ddH_2_O. Different concentrations of (NH_4_)_2_HPO_4_ (50, 100, and 150 mM, for Groups 1, 2, and 3, respectively) were added to the prepared alginate solution, which was then mixed using a planetary centrifugal mixer (ARM‐310; Thinky Corporation, Tokyo, Japan) for 5 min at 2000 rpm. The prepared LGG (Section 2.2) was added to each of the (NH_4_)_2_HPO_4_ and alginate solutions. The mixtures were extruded into 0.15 M CaCl_2_ solution at a rate of 2 mL/min using an auto‐injector (KD Scientific Inc., Holliston, Massachusetts). After 1 h of gelation, the HAp/Alg composite hydrogel beads were harvested and washed thrice with ddH_2_O. Alginate (Alg) hydrogels were prepared as controls.

### 2.4 LGG survival at gastric pH

Simulated gastric fluid was prepared as previously described.^22^ Briefly, 0.8 g NaCl was dissolved in 395 mL ddH_2_O. When the solution turned clear, the pH was adjusted to 2.0 using 1 M HCl, and ddH_2_O was added to a total volume of 400 mL. Each hydrogel sample (1 g), containing the encapsulated LGG, was added to the simulated gastric fluid. The hydrogels were incubated in the gastric fluid for 2 h (37°C, with shaking at 99 rpm). The hydrogels were degraded using a 10% citrate buffer. Encapsulated‐LGG survival was determined by plate counting. To visualize the enzymatic activity of the encapsulated LGG, the hydrogels were incubated with carboxyfluorescein diacetate (cFDA) dye and observed using an in vivo imaging system (IVIS).

### 2.5 Release of encapsulated LGG at intestinal pH

The intestinal pH solution was prepared by dissolving 2.72 g KH_2_PO_4_ in 385 mL ddH_2_O, the pH was adjusted to 7.2 using 1 M NaOH, and ddH_2_O was added to a total volume of 400 mL. Each hydrogel sample (1 g) was incubated in an intestinal pH solution for 4 h. Samples (100 μL) were collected at different time points (0, 1, 2, 3, and 4 h). The release of LGG was evaluated by plate counting. The proportion of LGG (expressed as a percentage) released from the hydrogel was calculated as follows:

$$\text{Released}\;\mathrm{LGG}\;\left(\%\right)=\frac{\text{Count of released}\;\mathrm{LGG}}{\text{Initial}\;\mathrm{LGG}\;\text{count}}\times 100$$

### 2.6 Determination of LGG survival

Viable LGG cells were counted using the plate counting method. After treatment, 100 μL of each sample was serially diluted (from 10^0^ to 10^−7^), plated onto MRS agar plates, and incubated at 37°C for 48 h under anaerobic conditions. Finally, the colonies were counted on MRS agar plates.

### 2.7 Examination of the efficacy of HAp/Alg composite hydrogels in mice

The procedures involving animals were performed according to the Pusan National University Institutional Animal Care and Use Committee (PNU‐IACUC) regulations, and were approved on February 24, 2021 (approval number: PNU‐2021‐2893). Male imprinted control region (ICR) mice (7 weeks old, 30–35 g) were used as the animal models. The mice were maintained with food and water ad libitum at 22°C ± 1°C with a 12 h light/dark cycle for 1 week before experimentation, to facilitate their adaptation.

#### 2.7.1 DSS‐induced colitis induction in mice

Colitis was induced in all mice, except in the healthy control group, via oral administration of 3% (wt/vol) DSS in distilled water ad libitum for 7 days. During the induction period, body weight and disease activity index (DAI) was assessed daily.^23^

#### 2.7.2 Therapeutic efficacy in the DSS‐induced colitis mouse model

Male ICR mice (7 weeks old) were divided into four groups (healthy, colitis, Alg, and HAp/Alg; five per group). Colitis was induced by administering 3% (wt/vol) DSS in drinking water, as described above. At the same time, the Alg and HAp/Alg groups received the same amount of LGG (~7 log CFU/bead) orally once every alternate day for 14 days. The healthy and colitis control groups received ddH_2_O instead of hydrogel beads. The clinical progression of colitis was monitored daily by assessing DAI.^24^

#### 2.7.3 Evaluation of colitis severity using DAI

DAI is an index reflecting weight loss (relative to initial body weight), stool consistency, and rectal bleeding, each scored from 0 to 4. Weight loss was scored as follows: 0, none; 1, 1%–5% weight loss; 2, 5%–10%; 3, 10%–20%; and 4, >20%. Stool consistency was scored as follows: 0, normal stool; 2, loose stool; and 4, diarrhea. Bleeding was scored as follows: 0, no bleeding; 2, bleeding; and 4, gross bleeding.

#### 2.7.4 Evaluation of colon length and spleen weight

On the last day of the experiment (Day 14), the mice were euthanized, and the entire colon, from the cecum to the anus and spleen, was dissected. The colon length and the weight of the spleen of each mouse were measured as indicators of colonic inflammation.^25^, ^26^

#### 2.7.5 Histological assessment of colitis

The severity of colitis was further evaluated via histological examinations.^27^ Colon samples were fixed in 10% formalin for 24 h and embedded in paraffin blocks. Colon tissue sections (5 μm thick) were cut using a microtome (Shandon, Pittsburgh, Pennsylvania). A cross‐section of the colon was stained with hematoxylin and eosin and/or alcian blue (according to the manufacturer's instructions with modifications) and observed under a light microscope (Olympus Corporation, Tokyo, Japan) to determine colon morphology, epithelial injury, degree of inflammation, and goblet cell count.^28^

### 2.8 Statistical analysis

Statistical analyses of all in vitro and in vivo data were performed using one‐way and two‐way analysis of variance (ANOVA), followed by the Bonferroni test, using GraphPad Prism software (v.5.0; GraphPad Software, Inc., LA Jolla, California). Differences were considered statistically significant at *p* < 0.05.

## 3 RESULTS AND DISCUSSIONS

### 3.1 Fabrication of the pH‐sustaining HAp/Alg composite hydrogel

**FIGURE 1:**
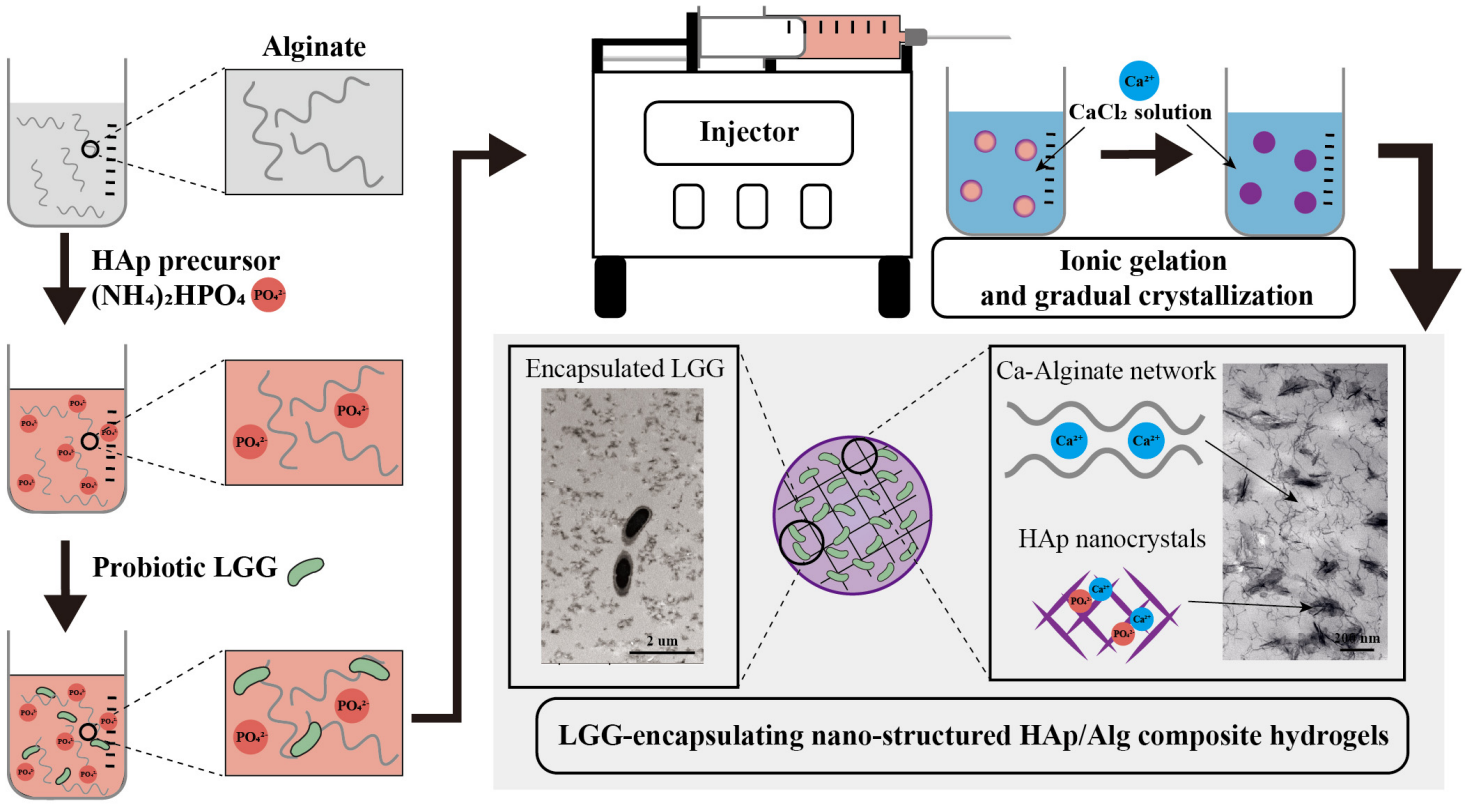
Fabrication of nanostructured hydroxyapatite/alginate (HAp/Alg) composite hydrogel. LGG, *Lactobacillus rhamnosus* GG.

**FIGURE 2:**
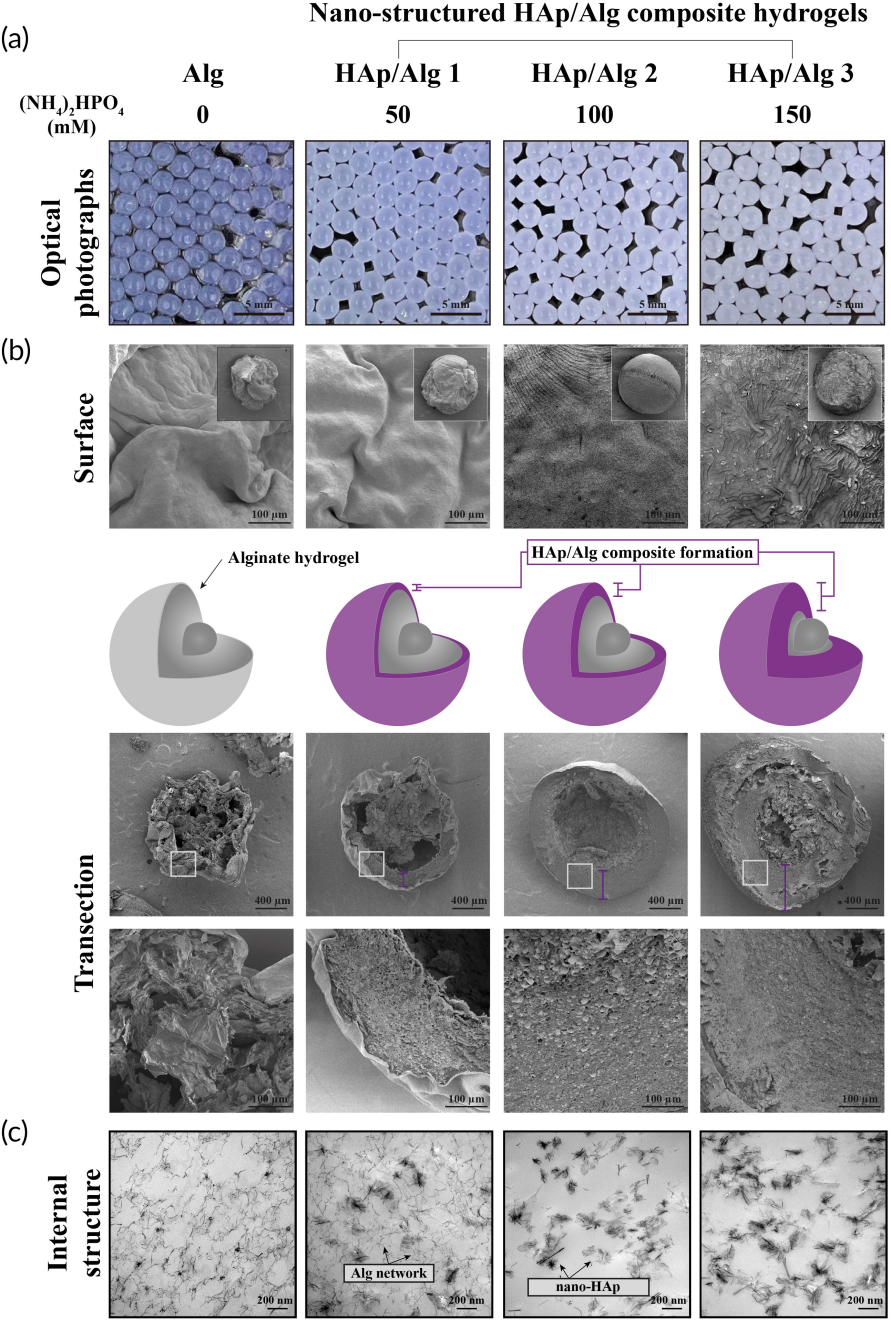
*Lactobacillus rhamnosus* GG (LGG)‐encapsulating nanostructured hydroxyapatite/alginate (HAp/Alg) composite hydrogels. (a) Optical photographs of HAp/Alg composite hydrogels with different concentrations of hydroxyapatite. (b) Surface and transection scanning electron microscopy images of HAp/Alg hydrogels, with a schematic. (c) Transmission electron microscopy images of nanosized HAp crystals and Alg hydrogel network.

An ideal probiotics delivery system should minimize loss of viability during fabrication by applying simple manufacturing processes, effectively protecting the encapsulated probiotics in a stomach, and releasing them in the intestine.^29^ Here, we fabricated the HAp/Alg composite hydrogel in one step, via extrusion of a diammonium hydrogen phosphate ((NH_4_)_2_HPO_4_) and Alg mixture into a calcium chloride solution (Figure 1). The Alg hydrogel was formed via the interaction between the calcium and the guluronic acid in the Alg polymer; the HAp crystallizes gradually from the source calcium and phosphate. The HAp/Alg composite hydrogel is white and opaque, whereas the Alg hydrogel is transparent and reflective (Figure 2a). As the HAp:Alg ratio increases, the composite hydrogel becomes a brighter white, more opaque, and less reflective. The HAp/Alg composite hydrogel formed beads of ~2.2 mm in diameter, with no significant size differences between the groups.

### 3.2 HAp/Alg composite hydrogel structure

To compare the HAp/Alg composite and bare Alg hydrogels, we analyzed hydrogel bead structure via electron microscopy, using SEM to analyze composite hydrogel bead surfaces and transections, and TEM to analyze the internal structure. While the surface and transection of the Alg hydrogel beads exhibited shrinkage and cracking, the HAp/Alg composite hydrogel beads maintained their shape (Figure 2b). When fabricating the HAp/Alg composite hydrogel beads, we generated three experimental groups, adding the precursor (NH_4_)_2_HPO_4_ at 50, 100, or 150 mM, to Groups 1, 2, and 3, respectively. The HAp/Alg composite hydrogel beads had a spherical shape and smooth surface. They exhibited characteristic regular patterns of crystallized HAp on the surface, particularly in HAp/Alg Groups 2 and 3, implying that HAp crystallized well within the Alg hydrogel network. Dense layers were observed in the HAp/Alg bead transections. In the HAp/Alg Group 3, most of the area in the system was filled with composite layers of HAp and Alg, while the Alg hydrogels exhibited shrinkage and cracking, with multiple voids. The TEM images (Figure 2c) reveal the internal structure of the HAp/Alg composite hydrogel beads. Nanosized HAp crystals were homogeneously dispersed in the Alg hydrogel network, and the number of the HAp crystals increased with increasing concentrations of the precursor (NH_4_)_2_HPO_4_. LGG encapsulation in the HAp/Alg composite hydrogel was verified as shown in Figure 1. In the result of FT‐IR (Figure S1), HAp/Alg composites were distinguished from Alg hydrogels based on the identification of phosphate peaks associated with HAp (550–650 cm^−1^).^30^ The characteristic peak of mannuronic acid in Alg (1088 cm^−1^) was diminished as the amount of HAp was increased, whereas the peak of guluronic acid (1030 cm^−1^) in Alg remained unaffected.^31^ The findings suggest that there is an interaction between the calcium ions in HAp and the mannuronic acids in Alg, whereas the guluronic acids in Alg is involved in the formation of calcium–Alg hydrogel networks.

### 3.3 Internal pH changes in HAp/Alg composite hydrogel beads under gastric conditions

**FIGURE 3:**
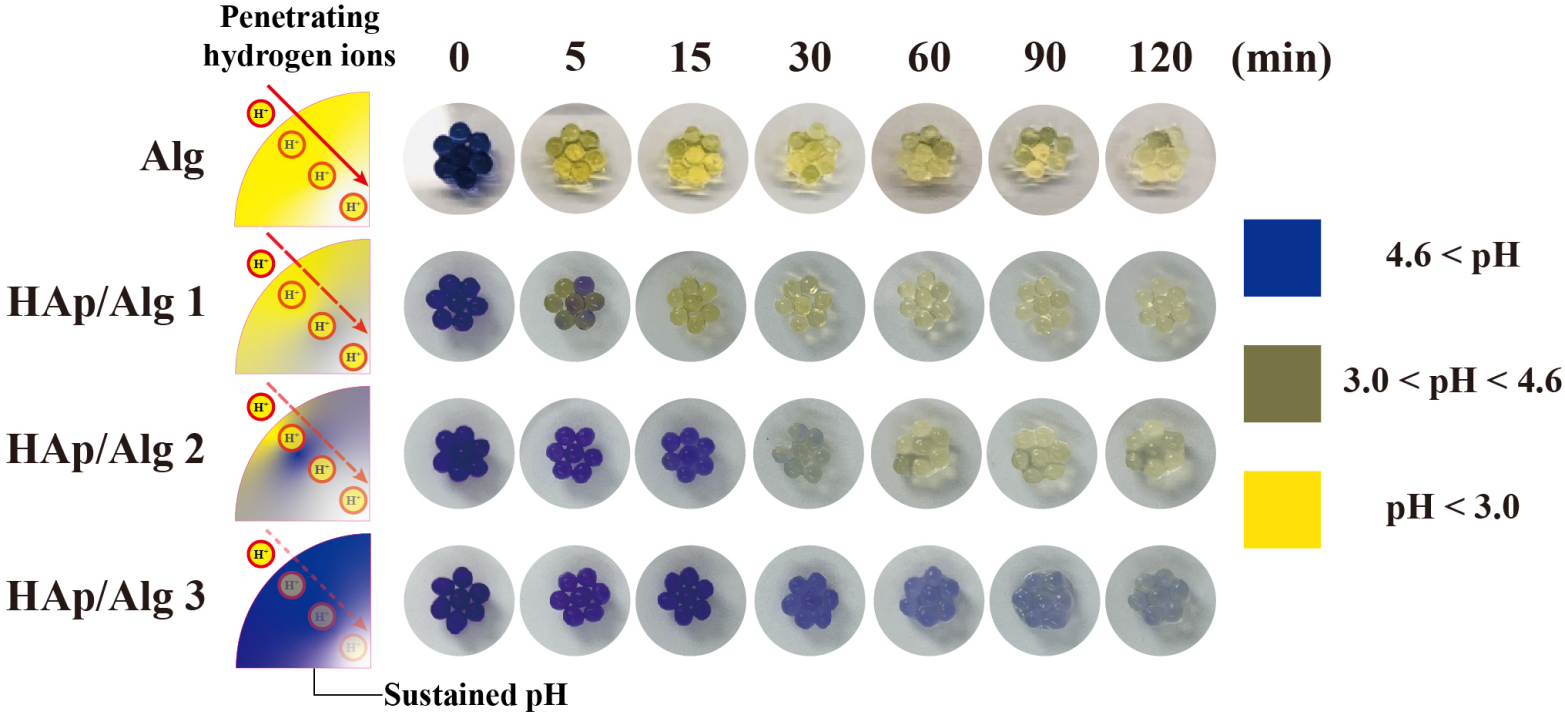
Changes in internal pH changes of hydroxyapatite/alginate (HAp/Alg) composite hydrogels in simulated gastric fluid. Colorimetric changes in bromophenol blue‐loaded HAp/Alg composite hydrogels at gastric pH with a schematic illustration.

Gastric pH ranges from 1.0 to 2.5 and threatens the survival of the administered probiotics, disturbing their membrane integrity and causing loss of enzymatic activity due to an excess of hydrogen ions.^32^, ^33^ The penetration of acidic gastric fluid into the highly porous hydrogel networks, and the subsequent drop in pH, presents a significant drawback of Alg‐based probiotic delivery systems. We sought to avoid this problem via our pH‐sustaining HAp/Alg composite hydrogel system. HAp maintains hydrogel pH by neutralizing hydrogen ions that penetrate the hydrogels (Figure 3). To verify pH maintenance in the HAp/Alg composite hydrogel during incubation at gastric pH, BPB‐loaded HAp/Alg hydrogel beads were incubated in simulated gastric fluid for 120 min. BPB is blue above pH 4.6 and turns yellow below pH 3.0. After 5 min, the Alg hydrogel turned yellow, consistent with a prior report.^6^ Changes in color were observed at 15, and 60 min in HAp/Alg Groups 1, and 2 respectively. The color of HAp/Alg 3 did not turn yellow, indicating that the microenvironmental pH around encapsulated LGG in the hydrogel was sustained under acidic pH conditions.

### 3.4 Gastric survival of LGG

**FIGURE 4:**
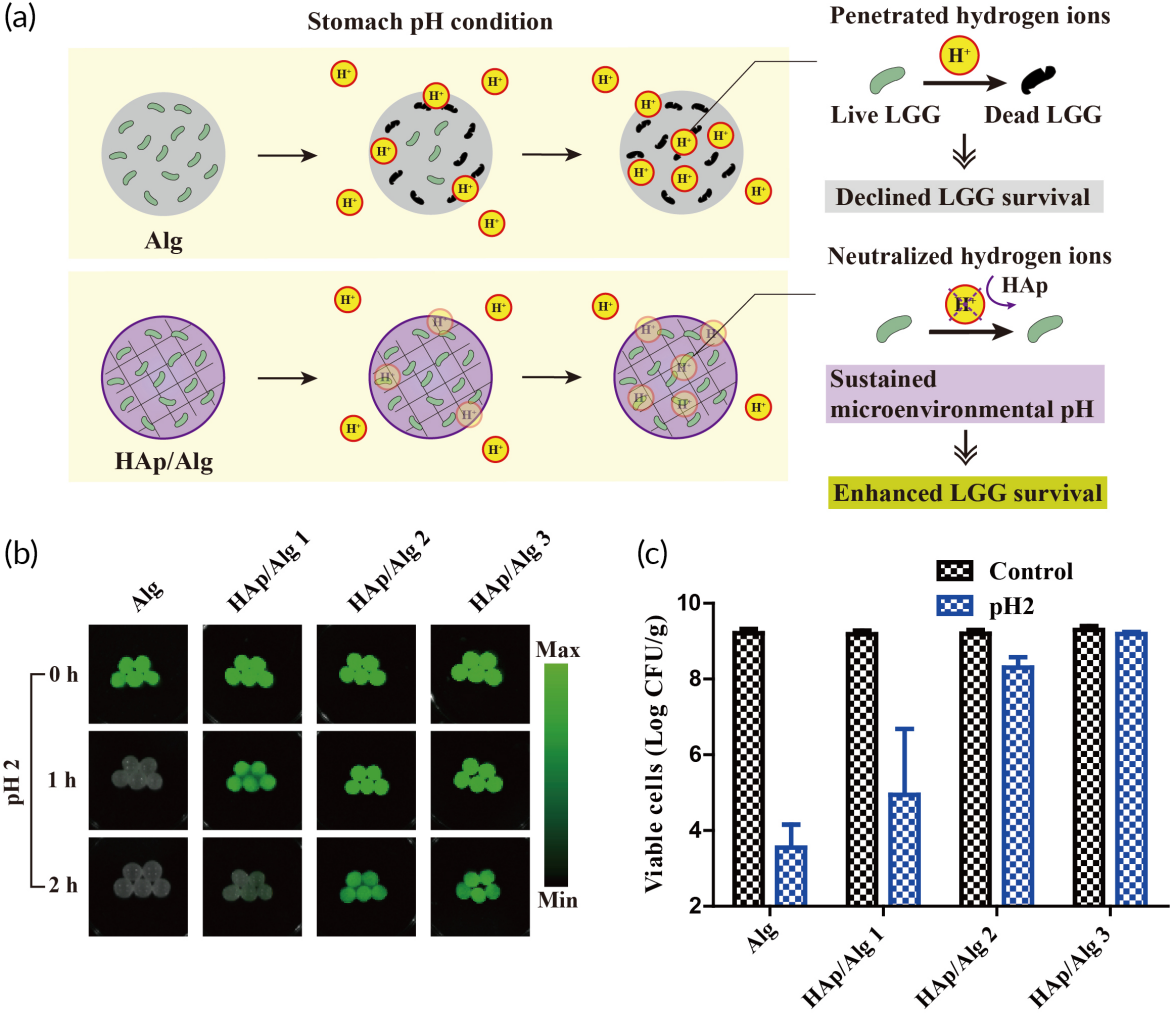
Survival of encapsulated *Lactobacillus rhamnosus* GG (LGG) after gastric pH challenge. (a) Schematic of pH maintenance by hydroxyapatite/alginate (HAp/Alg) composite hydrogels in the stomach. (b) The protective effect of these hydrogels, tracked using carboxyfluorescein diacetate‐labeled LGG during gastric pH challenge. (c) LGG survival following gastric pH challenge enumerated by plate counting.

Encapsulated probiotics require pH homeostasis in order to maintain their enzymatic function and to survive in a harsh low‐pH environment. Without this homeostasis, the survival of the encapsulated probiotics is threatened in a stomach pH condition (Figure 4a). We observed a sustained pH in the HAp/Alg composite hydrogel beads (Figure 3). We used cFDA, a fluorescent dye, to assess enzymatic activity of HAp/Alg composite hydrogel‐encapsulated LGG. CFDA is a lipophilic compound that penetrates the cytoplasm of microorganisms and causes fluorescence when the ester bond is cleaved by esterases. When LGGs lose their enzymatic activity due to detrimental pH conditions, cFDA should not induce fluorescence. Two hours after gastric pH treatment, the fluorescence intensity of the HAp/Alg composite hydrogel was maintained, while that of the Alg hydrogel beads was lost (Figure 4b).

To quantify the protective effect of the HAp/Alg composite hydrogel beads against gastric pH, we quantified LGG survival via plate counting (Figure 4c). The initial counts were not significantly different between the HAp/Alg composite and Alg hydrogels (9.22 ± 0.11 log CFU/g), implying that there was no loss of viability due to HAp crystallization. After treatment with simulated gastric fluid at pH 2, the survival of the LGG in the Alg hydrogels decreased sharply, to 3.55 log CFU/g. For the HAp/Alg composite hydrogel‐encapsulated LGG, survival increased gradually as the concentration of the precursor diammonium hydrogen phosphate increased. In HAp/Alg Group 3, 9.19 log CFU/g of the encapsulated LGG survived, similar to the initial count. Live probiotic counts >6–7 log CFU/g were required to achieve the desired clinical effects.^34^, ^35^ Based on their performance in the simulated gastric fluid, HAp/Alg composite hydrogels at precursor concentrations of 100 or 150 mM (Groups 2 and 3) are promising candidates for effective intestinal delivery system for LGG.

Further, we prepared pre‐made HAp powder containing alginate (HAp+Alg) hydrogels and its gastric LGG survival was compared with that of nanostructured HAp/Alg composite hydrogels to see if HAp nanocrystallization process and the HAp/Alg composite formation play an important role in protecting encapsulated LGG against gastric pH. Interestingly, HAp+Alg hydrogels showed much lower LGG survival (<log 7 CFU/g; Figure S2) under gastric pH conditions than nanostructured HAp/Alg composite hydrogel, demonstrating that homogenous dispersal of nanosized HAp crystals within Alg hydrogel network is critical to effectively maintain microenvironmental pH around the encapsulated LGG under gastric pH conditions.

### 3.5 Encapsulated‐LGG release profiles at intestinal pH

**FIGURE 5:**
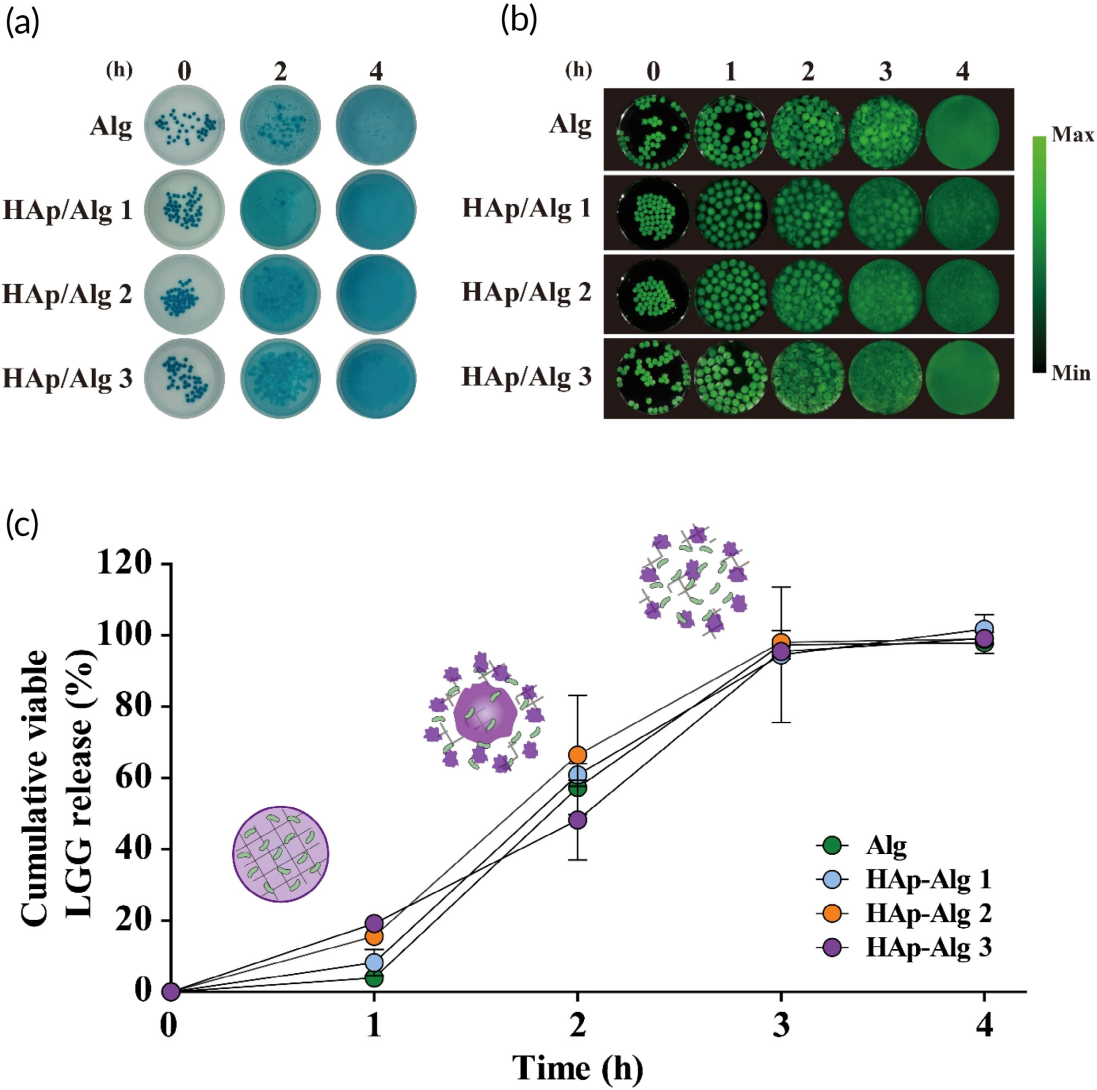
Hydroxyapatite/alginate (HAp/Alg) composite hydrogels at intestinal pH. (a) Composite hydrogel disintegration at intestinal pH was tracked via alcian blue staining. (b) Encapsulated‐*Lactobacillus rhamnosus* GG (LGG) release from the hydrogels. LGG was labeled with IR780 and observed via an in vivo imaging system at the indicated time points. (c) Encapsulated‐LGG release profile upon hydrogel disintegration with a schematic. Data represent the mean ± SD (*n* = 3).

Because orally administered probiotics have beneficial effects in the intestinal region, including the ileum and ascending colon, they should be released from the delivery system upon reaching the intestine in order to exert their beneficial effects. Here, encapsulated LGG was released upon disintegration of the HAp/Alg composite hydrogel at the intestinal pH (Figure 5). Disintegration of the hydrogel beads was tracked with alcian blue (Figure 5a). There was no significant difference in the disintegration rate, and after 4 h of incubation at pH 7.2, disintegration was complete in all four groups. To visualize LGG release at intestinal pH, IR780‐stained LGG was encapsulated in the hydrogels, which were then incubated at intestinal pH (Figure 5b). After 4 h of incubation, encapsulated LGG in all groups was completely released (Figure 5c).

At intestinal pH, the COOH groups of Alg are converted to COO^−^ and as a result of electrical repulsion, the Alg hydrogels swell and disintegrate. The patterns of disintegration and release during intestinal pH incubation did not differ between the HAp/Alg composite and Alg hydrogels. The formation of the HAp/Alg composite hydrogel did not inhibit the release of the encapsulated probiotics; therefore, its protective effect can be maximized by increasing the HAp concentration. In addition, the stability of encapsulated LGG was assessed for 4 weeks at 4 °C (Figure S3). All groups maintained viable counts above 8 log CFU/g. The HAp/Alg groups showed a slight increase in the viable counts of LGG in comparison to the Alg group, possibly due to the neutralization of lactic acid produced by LGG.

### 3.6 Visualization of HAp/Alg composite hydrogel gastrointestinal behavior in a mouse model

**FIGURE 6:**
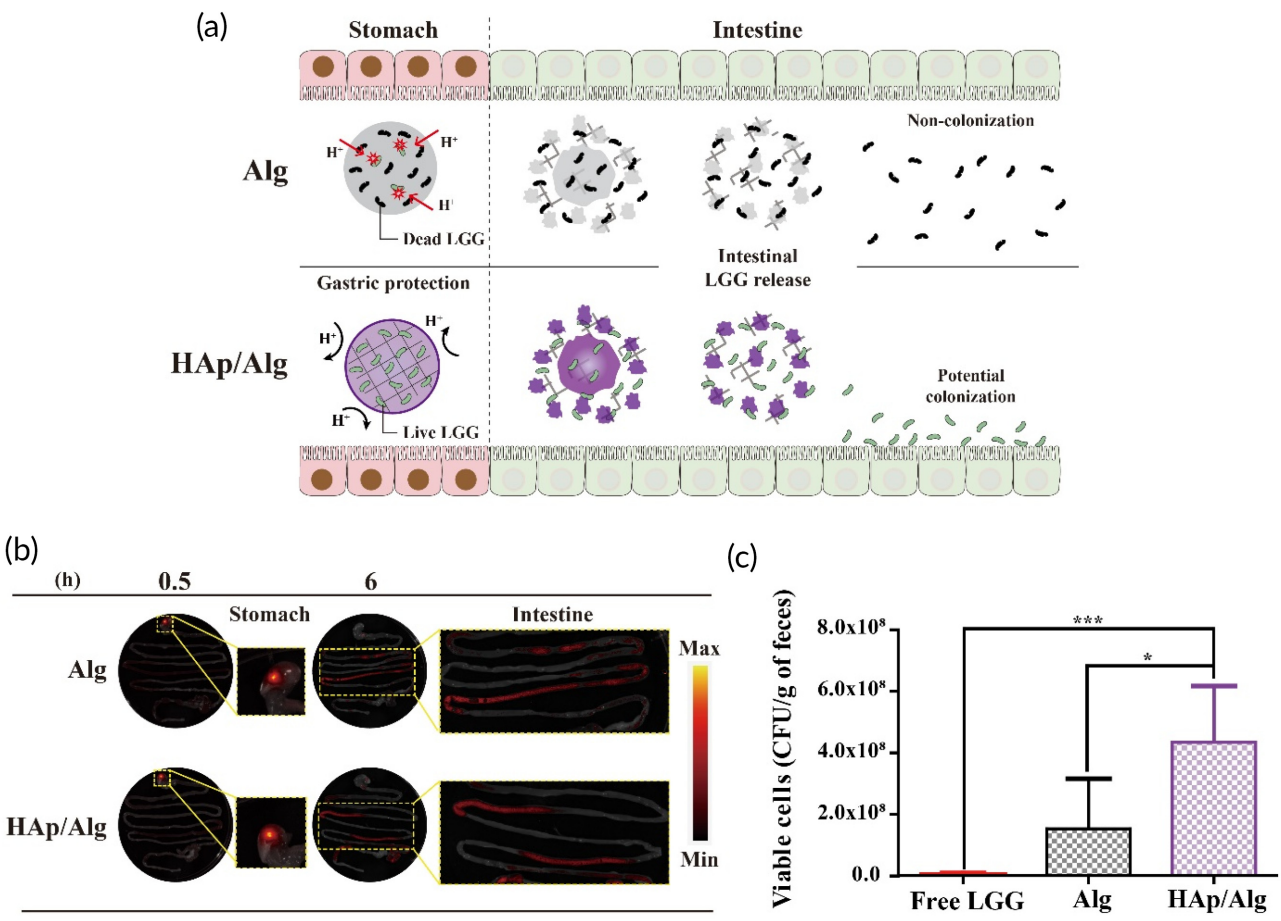
Gastrointestinal activity of hydroxyapatite/alginate (HAp/Alg) composite hydrogel and Alg hydrogel in mice. (a) Schematic of HAp/Alg composite hydrogel gastrointestinal activity. (b) IR780‐stained *Lactobacillus rhamnosus* GG (LGG) was encapsulated within the hydrogels and visualized using an in vivo imaging system. The gray rectangles comprising indicate the presence of LGG (red) in the gastrointestinal tract. (c) Fecal recovery of LGG 24 h after oral administration. **p < 0.01.

For efficient intestinal delivery of probiotics, the delivery system should ideally protect the encapsulated probiotics in the stomach, maintaining intact scaffolds, and release the probiotics at a target area in the intestine. To observe the gastrointestinal behavior of the hydrogels encapsulated‐LGG, IR780‐stained LGG was encapsulated in the HAp/Alg composite and Alg hydrogels, and single beads were administered to each mouse (Figure 6b). Thirty minutes after oral administration, the beads retained their spherical shape in the stomach for both hydrogels. Peristalsis and monovalent ion often cause disruption in Alg hydrogel^13^, ^36^; however, there was no premature release of LGG from both hydrogels. The intact shape of Alg hydrogel in the stomach was possibly due to prolonged gelation time during a manufacturing process. Although the HAp/Alg composite hydrogel potentially has reinforced greater mechanical stability than the Alg hydrogel^37^ owing to its denser structure and pH‐triggered Ca^2+^ ion replenishment, there was no observable difference in bead shape in the stomach. Six hours after administration, the beads of both hydrogels had completely disintegrated in the intestines. Under in vitro and in vivo conditions, the hydrogels remained intact in the stomach, ensuring protection from the low pH, and the encapsulated LGG was completely released upon hydrogel disintegration (Figure 6b). Furthermore, fecal recovery after 24 h of oral administration was performed to observe the colonization by LGG (Figure 6c). Viable LGG counts from the HAp/Alg composite hydrogel were 2.86 times greater than those from the Alg hydrogel. Overall, the composite formation of HAp/Alg in the hydrogel did not inhibit LGG release, allowing the LGG to persist in the intestines for at least 24 h.

### 3.7 The therapeutic effect on DSS‐induced ulcerative colitis: Macroscopic evaluation

**FIGURE 7:**
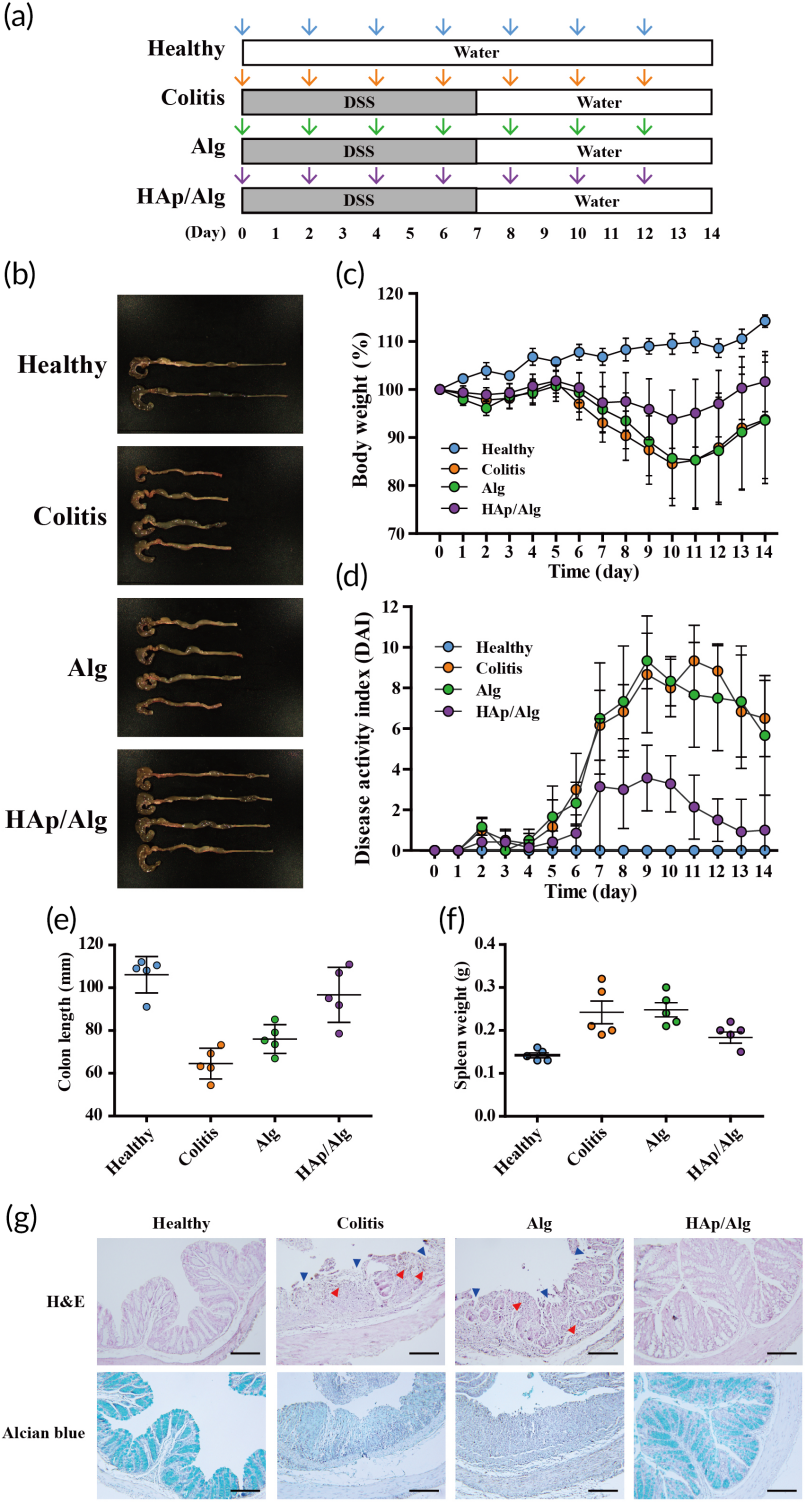
Therapeutic effects on ulcerative colitis of *Lactobacillus rhamnosus* GG (LGG) encapsulating hydroxyapatite/alginate (HAp/Alg) composite hydrogels. (a) Experimental scheme of colitis induction and hydrogel administration in dextran sulfate sodium‐induced colitis. (b) Representative photograph of the colon from each of the experimental groups. (c) Change in body weight (%). (d) Disease activity index. (e) Colon length. (f) Spleen weight (*n* = 5). The results are presented as the mean ± SD. (g) Hematoxylin and eosin (H&E) staining and alcian blue staining of colon sections. Blue triangles indicate disrupted epithelium and red triangles indicate inflammatory cell infiltration. The scale bar represents 200 μm.

To observe their therapeutic effects, we administered a HAp/Alg hydrogel bead orally in a DSS‐induced ulcerative colitis mouse model. Colitis was induced by providing DSS‐containing water for 7 days, and the hydrogels were administered every second day for 14 days (Figure 7a). We selected the DSS model mice based on DSS‐induced colitis histopathology, which resembles colitis histopathology in humans. The DSS model and healthy control groups did not differ in body weight during the adaptation period. Unlike the healthy control group, body weight of mice was reduced in the colitis, Alg, and HAp/Alg groups after colitis induction. After DSS administration ceased, body weight recovered more rapidly in the HAp/Alg group than in the colitis and Alg groups (Figure 7c). Colitis clinical progression was monitored daily by assessing the DAI.^24^ DAI was constant in the healthy control group, indicating that no colitis was induced. On Day 10 after colitis induction, the colitis and Alg groups both exhibited severe bleeding and diarrhea, reflected in the high DAI scores (Figure 7d), although DAI was lower in the HAp/Alg group than in the Alg group. Colon shortening, accompanied by severe bleeding and diarrhea, frequently occurs in DSS‐induced ulcerative colitis.^23^ Colon length was significantly shorter following ulcerative colitis induction than in the healthy control group. The average colon lengths were 106.13, 64.56, 76.02, and 96.68 mm, in the healthy control, colitis, Alg, and HAp/Alg groups, respectively (Figure 7e), indicating that the composite hydrogel ameliorated the colitis symptoms. We used an increase in the weight of the spleen, an essential organ in the immune system, as a marker of inflammation severity (Figure 7f). Spleen weight was significantly higher in the colitis and Alg groups than in the HAp/Alg groups. Overall, the LGG‐encapsulated HAp/Alg composite hydrogels ameliorated ulcerative colitis, as indicated by the changes in body weight, DAI, colon length, and spleen weight.

### 3.8 Therapeutic effect on DSS‐induced ulcerative colitis: Histological analysis

To assess the therapeutic effect of the HAp/Alg composite hydrogel on ulcerative colitis, we performed histological examination of colon tissue via hematoxylin and eosin staining and alcian blue staining. In the healthy control group, the colon sections exhibited no evidence of disrupted epithelium, inflammatory cell infiltration, or mucosal edema (Figure 7g). The colitis and Alg groups exhibited no notable improvement in colitis, with evidence of severe colitis, including epithelial damage, submucosal edema, and infiltration by inflammatory cells (neutrophils and macrophages). In contrast, the HAp/Alg group exhibited substantial improvement, with re‐epithelization and histology resembling that of healthy colon sections.

Ulcerative colitis is accompanied by defective colonic mucus layers and depletion of goblet cells (mucin‐secreting intestinal cells).^38^ To observe the recovery of the intestinal barrier, we stained the goblet cells with alcian blue (Figure 7g). Goblet cell numbers were reduced in the colitis and Alg groups but were similar in the healthy and HAp/Alg groups. Hence, the intestinal delivery of LGG, with minimal loss of enzymatic functions and viability, ameliorated colitis, as indicated by changes in epithelial damage, submucosal edema, inflammatory cell infiltration, and the number of goblet cells. Overall, the enhanced therapeutic efficacy of the HAp/Alg composite hydrogel can be attributed to the sustained microenvironmental pH around the encapsulated LGG, which is due to the pH‐buffering property of HAp and the composite formation of nanostructured HAp/Alg as demonstrated in Figures 2, 3, 4, 7, and S2. Notably, the HAp/Alg hydrogel offers a clear advantage in achieving full gastric pH protection while maintaining the release of live microorganisms in the intestine and providing a simple fabrication process.

## 4 CONCLUSIONS

With the increasing efforts to utilize live microorganisms for therapeutic purposes, the demand for effective delivery systems has increased. To address this, we developed an HAp/Alg composite hydrogel for the intestinal delivery of LGG. We fabricated the HAp/Alg composite hydrogels via a one‐step ionic Alg gelation and HAp crystallization process. SEM revealed characteristic crystallization patterns on the composite hydrogel bead surfaces, and TEM revealed nanostructured HAp crystal formation in the Alg polymer. The HAp crystals were homogeneously dispersed, and LGG encapsulation was confirmed. At gastric pH, the HAp/Alg composite hydrogel beads maintained their internal pH for 2 h, effectively protecting the encapsulated LGG. At intestinal pH, the composite HAp/Alg layers did not inhibit LGG release. Following oral administration, LGG‐encapsulated HAp/Alg composite hydrogel activity was tracked via fluorescence‐labeling of LGG and in vivo imaging. The HAp/Alg composite hydrogel beads remained intact in the stomach, and the LGG was completely released upon hydrogel disintegration in the intestine. Fecal recovery of LGG was higher in the HAp/Alg group than in the Alg group, implying that the composite hydrogel achieved better LGG viability. Administration of LGG‐encapsulating HAp/Alg composite hydrogel beads ameliorated colitis, as indicated by changes in body weight, DAI, colon length, spleen weight, and intestinal barrier properties. In summary, the pH‐sustaining nanostructured HAp/Alg composite hydrogel achieved gastric protection and intestine‐targeted release of LGG. These findings suggest that this composite system can achieve successful intestinal delivery of live microorganisms and ameliorate colitis.

## AUTHOR CONTRIBUTIONS

**Jihyun Kim:** Conceptualization (equal); data curation (equal); formal analysis (equal); investigation (equal); methodology (equal); writing – original draft (lead). **Shwe Phyu Hlaing:** Conceptualization (equal); data curation (equal); formal analysis (equal); investigation (equal); methodology (equal); software (equal); visualization (equal); writing – original draft (equal); writing – review and editing (equal). **Juho Lee:** Formal analysis (equal); methodology (equal). **Dongmin Kwak:** Formal analysis (equal); methodology (equal). **Hyunwoo Kim:** Formal analysis (equal); methodology (equal). **Aruzhan Saparbayeva:** Formal analysis (equal); methodology (equal). **In‐Soo Yoon:** Formal analysis (equal); methodology (equal). **Eunok Im:** Formal analysis (equal); methodology (equal). **Yunjin Jung:** Formal analysis (equal); methodology (equal). **Jin‐Wook Yoo:** Conceptualization (lead); funding acquisition (equal); project administration (equal); supervision (equal); writing – original draft (equal); writing – review and editing (lead).

## FUNDING INFORMATION

This work was supported by the National Research Foundation of Korea (NRF) grant funded by the Korea government (MSIT) (No. NRF‐2022R1A2C2004340).

## CONFLICT OF INTEREST STATEMENT

The authors have no conflicts of interest to declare.

## Supporting information


**Data S1:** Supporting information.

## Data Availability

The data that support the findings of this study are available from the corresponding author upon reasonable request.
