# Supplementary material for: pH‐sustaining nanostructured hydroxyapatite/alginate composite hydrogel for gastric protection and intestinal release of Lactobacillus rhamnosus GG
Source: Bioeng Transl Med. 2023 Apr 19;8(3):e10527. doi: 10.1002/btm2.10527 (PMC10189427; doi:10.1002/btm2.10527)
Supplement: Supplementary file 1 — Data S1: Supporting information. [file BTM2-8-e10527-s001.docx]

**Supplementary Information**

pH-sustaining nano-structured hydroxyapatite/alginate composite hydrogel for gastric protection and intestinal release of *Lactobacillus rhamnosus* GG

*Jihyun Kim^a,‡^, Shwe Phyu Hlaing^a,‡^, Juho Lee, Dongmin Kwak^a^, Hyunwoo Kim^a^, Aruzhan Saparbayeva^a^, In-Soo Yoon^a^, Eunok Im^a^, Yunjin Jung^a^, and Jin-Wook Yoo^a,*^*

*^a^*College of Pharmacy and Research Institute for Drug Development, Pusan National University, Busan 46241, Republic of Korea

^‡^Contributed equally to this manuscript.

***Corresponding Author: [jinwook@pusan.ac.kr](mailto:jinwook@pusan.ac.kr)

**Supplementary Methods**

*1. Structural characterization of HAp/Alg composite hydrogels*

Fourier transform infrared spectroscopy (FT-IR) spectra of HAp/Alg composite and Alg hydrogels were determined using a Nicolet iS 50 FT-IR spectrometer (Thermo Fisher Scientific, Indianapolis, IN, USA). The morphology of the hydrogels was assessed using a scanning microscope (SEM, Supra 25, Carl Zeiss, Jena, Germany).

*2. Preparation of pre-made hydroxyapatite containing alginate hydrogel*

To calculate the amount of hydroxyapatite in HAp/Alg 3, HAp/Alg 3 and Alg were freeze-dried for two days and weighed to calculate the amount of hydroxyapatite in HAp/Alg 3. Hydroxyapatite was prepared with (NH_4_)_2_HPO_4_ and CaCl_2_ solutions, then added to the alginate solution. The pre-made hydroxyapatite containing alginate hydrogels (HAp+Alg) were harvested after 1 h of gelation and washed thrice with ddH2O.

*3. Evaluation of LGG stability in hydrogel*

LGG-encapsulated hydrogels were stored at 4 °C for four weeks, and the hydrogel samples were collected at different time points (0, 1, 2, 3, and 4 weeks). The collected samples were disintegrated using 10% citrate buffer, and LGG viability was determined via plate counting.

**Supplementary Figures**

**
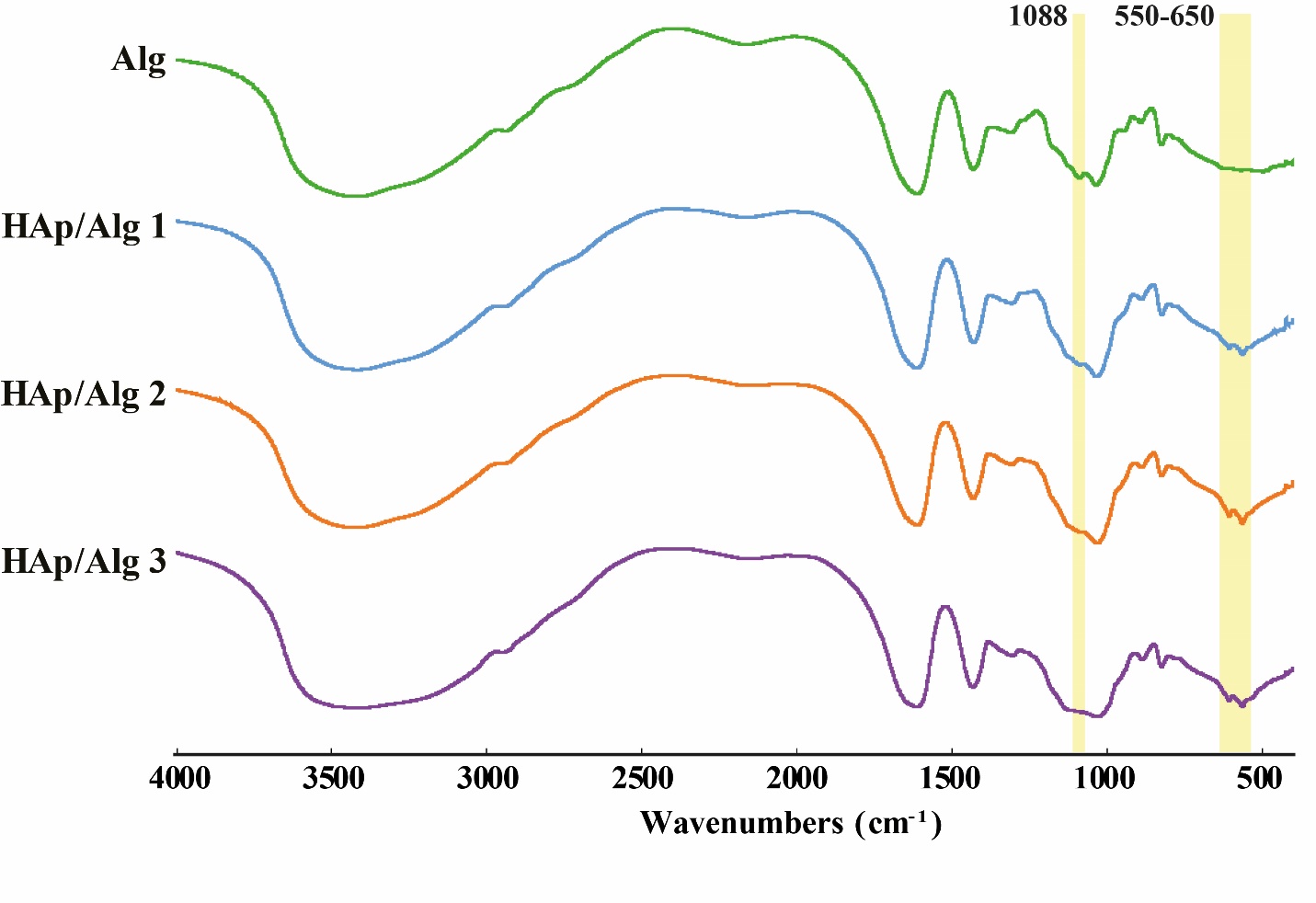
**

**Figure S1**. **FT-IR spectrum of HAp/Alg composite and Alg hydrogels.**

**
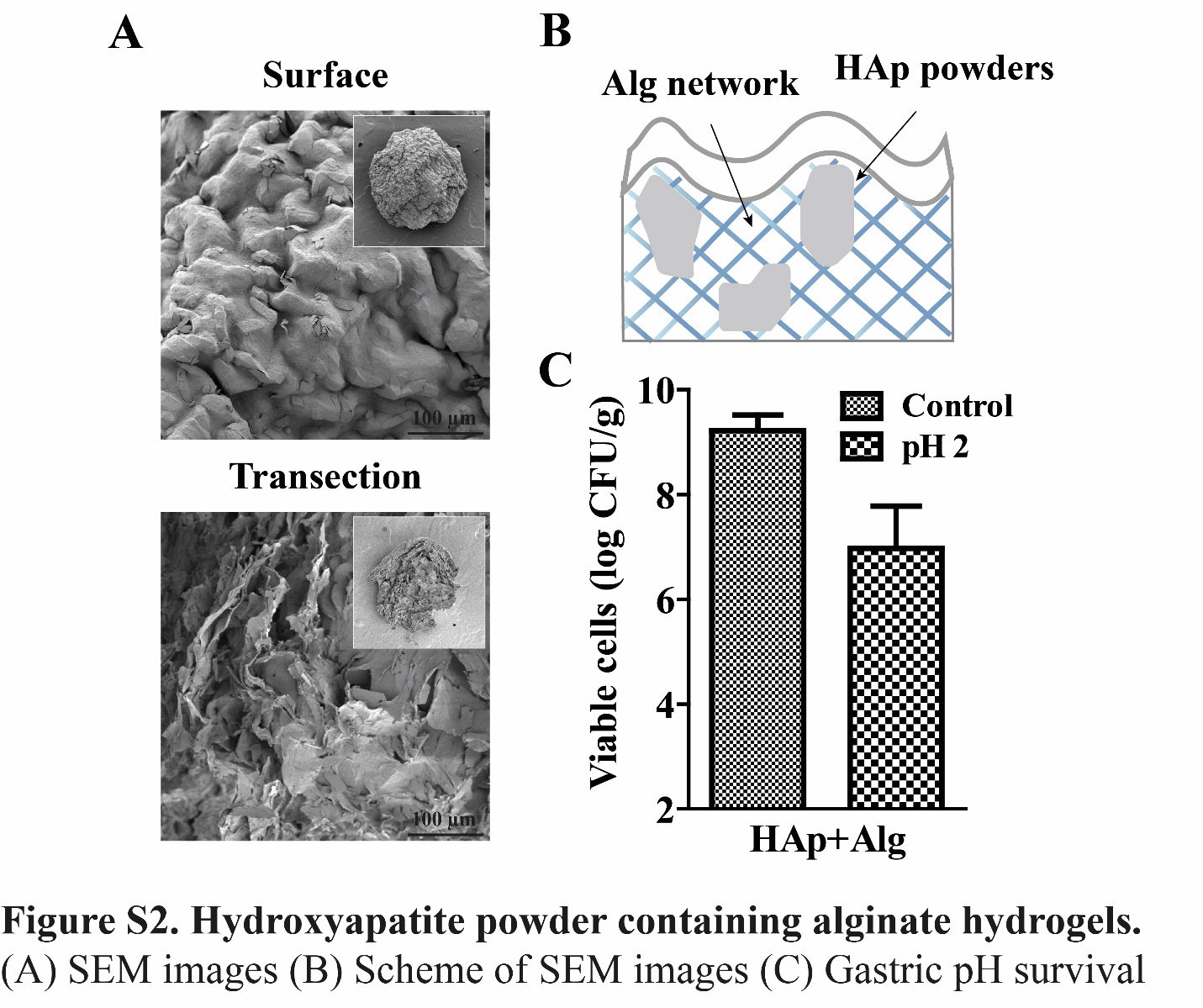
**

**Figure S2. Pre-made hydroxyapatite containing alginate hydrogels.** (A) SEM images. (B) Scheme of internal structures. (C) Gastric pH survival of encapsulated LGG.


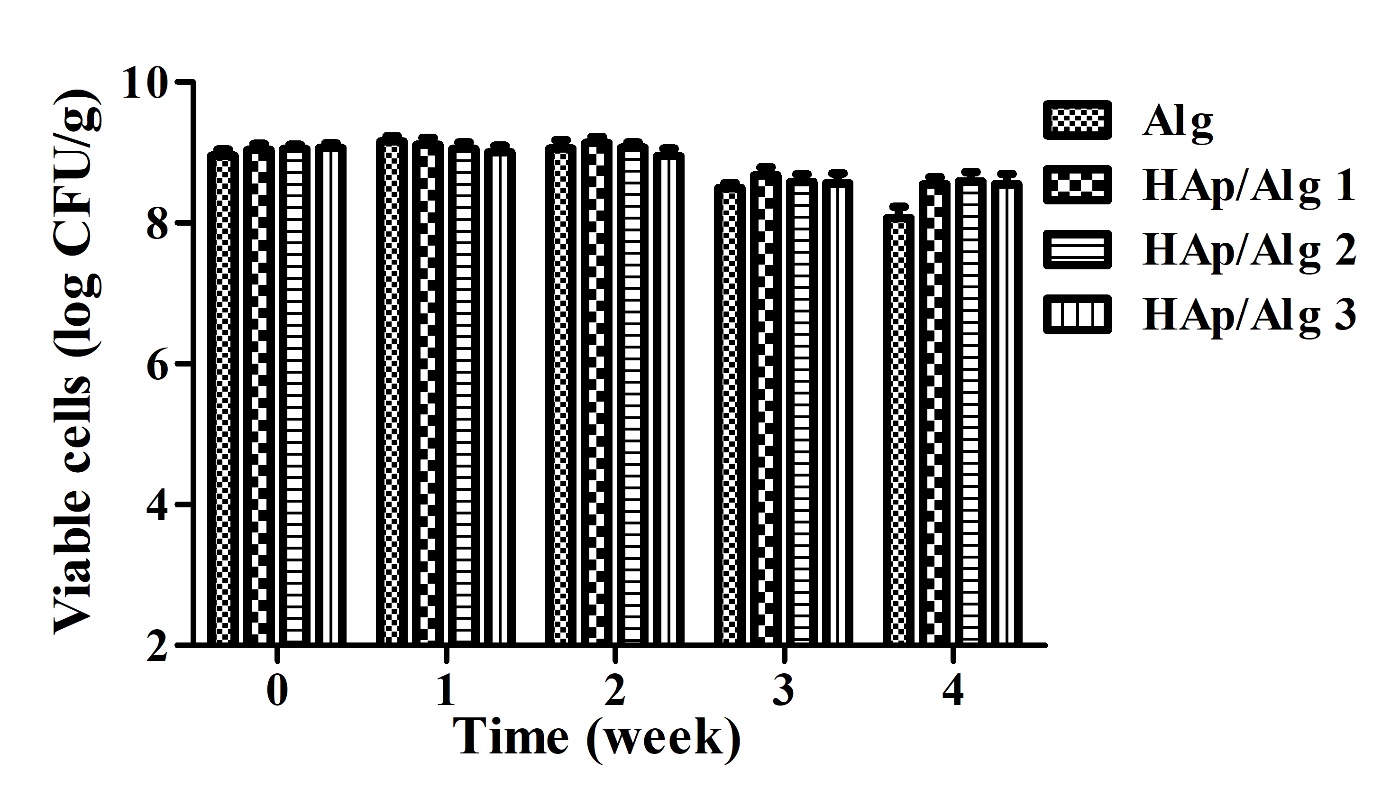


**Figure S3. Storage stability of HAp/Alg composite hydrogels at 4 °C.**
